# Viscoelastic Analysis of Asphalt Concrete with a Digitally Reconstructed Microstructure

**DOI:** 10.3390/ma17102443

**Published:** 2024-05-18

**Authors:** Marek Klimczak

**Affiliations:** Faculty of Civil Engineering, Cracow University of Technology, Warszawska 24 Street, 31-155 Cracow, Poland; marek.klimczak@pk.edu.pl

**Keywords:** asphalt concrete, viscoelasticity, Burgers model, image processing, finite element method

## Abstract

In the finite element analysis of asphalt concrete (AC), it is nowadays common to incorporate the information from the underlying scales to study the overall response of this material. Heterogeneity observed at the asphalt mixture scale is analyzed in this paper. Reliable finite element analysis (FEA) of asphalt concrete comprises a set of complex issues. The two main aspects of the asphalt concrete FEA discussed in this study are: (1) digital reconstruction of the asphalt pavement microstructure using processing of the high-quality images; and (2) FEA of the asphalt concrete idealized samples accounting for the viscoelastic material model. Reconstruction of the asphalt concrete microstructure is performed using a sequence of image processing operations (binarization, removing holes, filtering, segmentation and boundaries detection). Geometry of the inclusions (aggregate) are additionally simplified in a controlled mode to reduce the numerical cost of the analysis. As is demonstrated in the study, the introduced geometry simplifications are justified. Computational cost reduction exceeds of several orders of magnitude additional modeling error occurring due to the applied simplification technique. Viscoelastic finite element analysis of the AC identified microstructure is performed using the Burgers material model. The analysis algorithm is briefly described with a particular focus on the computational efficiency aspects. In order to illustrate the proposed approach, a set of 2D problems is solved. Numerical results confirm both the effectiveness of the self-developed code and the applicability of the Burgers model to the analyzed class of AC analysis problems. Further research directions are also described to highlight the potential benefits of the developed approach to numerical modeling of asphalt concrete.

## 1. Introduction

Asphalt concrete (AC) is one of the typical asphalt mixtures used in road engineering. It comprises aggregate, mastic (binder mixed with a filler) and a variety of possible additives and/or admixtures.

In traditional asphalt concrete, the weight ratio of mineral aggregate is more than 90%. Crushed stones (instead of natural ones) are used due to their better performance, relying on the superior binder to aggregate adhesion. Gradation curves of asphalt concrete can be easily distinguished from other mixture types. The content of each aggregate fraction is very similar (unlike for the stone mastic asphalt, SMA, for instance). Gradation curves of asphalt concrete are called “continuous” ones, which means that there is no missing aggregate fraction.

Inclusion particles can be classified as irregularly shaped and randomly distributed. It refers to both the spatial location and the angular orientation.

The overall mechanical properties of asphalt concrete, a heterogeneous material, are influenced by the mechanical properties of its constituents and their compatibility. Aggregate used for asphalt concrete is typically supplied from the local resources to reduce the transport costs. Consequently, various rocks (e.g., dolomite, basalt) can be used for this purpose. From the numerical perspective, aggregate behavior is usually modelled as linear elastic. Modeling of the bitumen response plays a dominant role in the whole numerical modeling process. This is due to a thermo-rheological mode of its performance. Practically, determination of both asphalt concrete and bitumen mechanical properties is performed at a specific temperature. This is the same for applied material model parameters. Some simplified approaches based on shift factors are also developed to speed-up the numerical modeling.

Heterogeneity of AC internal structure can be observed at manifold scales. In this study, the focus is on the biphasic material, as observed at the scale of asphalt mixture. Namely, we analyze the presence (and influence) of aggregate and binder phases. Precisely, the binder phase is, in fact, the mixture of mastic and fine aggregate. Lower scale(s) analyses are generally beyond the scope of this study. They are mentioned, however, when selected methods of AC internal structure recognition are described.

In some studies, the term “microstructure” is used specifically to describe the material internal structure observed at the scale accounting for the inclusions of dimensions of several μm [1,2]. On the other hand, it is also commonly used in multiscale analysis to describe the scale of the observed heterogeneity. Precisely, the overall material response is studied at a scale called the macroscale, and the underlying scale is called the microscale. In the case of a three-step multiscale analysis, a macroscale–mesoscale–microscale sequence is used. In this paper, the term “microstructure” refers to the scale of heterogeneity with the inclusions of dimensions of orders of mm.

Development in material sciences has been based on two main pillars, i.e., experiments and theoretical considerations, for centuries. It was the second half of the 20th century when numerical analysis became the third pillar of the advanced materials development process. Increasing availability of computational resources enabled researchers to carry out numerical analyses at a set of low scales. Such analyses turned out to be specifically helpful in the context of highly heterogeneous materials. It is possible to study the macroscale material response as a function of inclusions ratio, their orientation, location, angularity and other characteristics.

**Figure 1:**
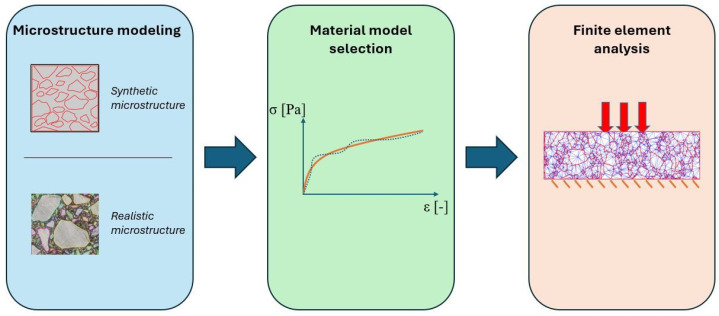
Scheme of heterogeneous materials finite element analysis on the example of AC.

Schematically, the finite element analysis of heterogeneous materials (at a specified scale) is shown in Figure 1. The first step is to model the internal structure of the material (AC, herein). Basically, two approaches are possible. One of them is to create a virtual/synthetic microstructure that possesses selected properties (e.g., the gradation curve and weight ratio) of the real material. A group of studies using this approach can be found in the literature [3,4,5,6,7,8,9]. Apparently, they may be considered as over-simplifying the real AC microstructure, thus are rather impractical. Their main advantage is the fact that the process of geometry generation can be easily parametrized. This gives the researcher the chance to study the performance of the specimens with different combinations of the controlled parameters. To some extent, such simulations can replace costly and time-consuming laboratory experiments when designing an optimal asphalt mixture.

An alternative approach to geometrical modeling of AC microstructures for further finite element analysis purposes is to digitally reconstruct the real specimen. In [10], three main methods are specified as digital camera imaging, scanning electron microscope (SEM) imaging and X-ray computed tomography (XRCT) scans. They are all classified as non-destructive testing (NDT) methods, which means (in the FEA context) that the analyzed specimen is not destroyed, and it can be used for laboratory experiments. Their results are of particular importance for the material model validation and calibration. A comprehensive review of these three methods is presented in [3]. Therein, the application limits of each of them are specified, to mention only one of the discussed aspects.

It should also be remarked that other numerical methods, besides the finite element method, are employed to study the performance of asphalt concrete. One of commonly used methods is the discrete element method. Its application in studying the performance of the asphalt concrete samples can be found in [2,9].

In our study, we use the digital camera imaging with further digital image processing (DIP). The choice of this technique can be explained in a manifold manner. Despite being a well-established method with a variety of successful applications, the scanning electron microscope is very limited in the spatial dimensions of the analyzed specimens. In [3], the maximum dimensions were specified as 50 mm (diameter of disc-shaped slices) or 50 mm × 50 mm (prismatic sample) with a maximum thickness of 10 mm. Thus, for the analysis purely at the asphalt mixture scale, this method is rather impractical. Application of this method of AC internal structure recognition is profitable in the case of lower scale analysis, such as the fracture mechanism due to adhesion failure between coarse aggregate and mastic [10], binder aging [11], adsorption of asphalt binder by aggregate [12], and self-healing properties of various additives [13], to mention only a few. Since the goal of this paper is the finite element analysis of the realistic sample (similar to these used in the laboratory experiments) dimensions, the scanning electron microscope imaging was eliminated in this specific application.

X-ray computed tomography scans and digital camera imaging (both followed by the digital image processing) are also well-established methods for AC internal structure recognition. As stated in [3], these techniques have been successfully employed to asphalt concrete internal structure recognition [14,15,16,17,18,19,20,21,22], to mention only a few of corresponding studies.

X-ray computed tomography scans have been used to reconstruct the AC microstructures for further finite [17,23] or discrete element analysis [24], but most of the studies are devoted solely to the analysis of the morphology [18,19,20,21,22] as a standalone problem. Seemingly, both applications need the same input, i.e., geometry of the microstructure. The difference is the graphics format. In the case of the morphology analysis (or discrete element analysis), raster graphics is sufficient. In the case of finite element analysis, a vector graphics description is required. Straightforward transform (as demonstrated in [25]) from raster graphics, by simple scaling pixels to physical dimensions, leads to the overkill finite element meshes and makes FEA excessively time consuming or even infeasible.

In [25], several algorithms of the microstructure geometry simplification were developed. In the present study, one of them (called local geometry enhancement) is employed. Simplification of the geometry is crucial for the efficiency of the further finite element analysis. Without this enhancement, only relatively small specimens could be analyzed, or the analysis time would be unacceptable for practical applications. What is important is that geometry simplification is an iterative but controlled process. Namely, the user can provide a set of conditions that need to be fulfilled before the iterations stop. It is due to the necessity of the aggregate shape preservation. It should be noted that the effectiveness of such an approach was demonstrated in [25]. Therein, the local geometry enhancement algorithm enabled the total number of degrees of freedom reduction of about 90%, with a corresponding additional modeling error not exceeding 0.1%.

In the present study, a self-developed framework for the AC viscoelastic finite element analysis with the reconstructed material internal structure is presented. As far as the method of the microstructure recognition is concerned, for illustrative purposes, high-quality digital image processing is used. This technique is typically used only for AC surface characterization, but these finding allow for various generalizations corresponding with the fully tridimensional internal structure [14,21]. For instance, an excellent fit between real gradation curves and those obtained from cross-sectional digital images could be obtained [14]. Alternatively, the cylinder side surface image was used to obtain a similar result [21]. Herein, high-quality digital image processing is used in order to illustrate the potential of the whole framework with emphasis on the numerical efficiency. Thus, the local geometry enhancement algorithm is employed to simplify the AC microstructure geometry in a controlled manner. Computational resources savings would be much more distinct in a 3D case with X-ray computed tomography (XRCT) scans processed in order to reconstruct the AC microstructure. This study is to illustrate the proposed approach, which is to be developed to the 3D case. At the current stage, it can be regarded as the processing of a single XRCT scan. Full tridimensional reconstruction would consist of merging a set of such slices into one geometry model.

Referring to Figure 1, the step following the microstructure geometry modeling in the finite element analysis workflow is the material model selection. As noted above, AC is considered in this study as a biphasic material, i.e., only two material phases are distinguished—binder and coarse aggregate (>2 mm inclusions). It is very common in the literature for aggregate to be modeled as linear elastic [6,7] and temperature-independent. Material models for the binder (and also for the adhesion between aforementioned phases) are of particular importance. A comprehensive review of the developed material models can be found in [4,26,27,28,29]. A detailed review is beyond the scope of this paper; however, some general research directions should be highlighted before the presentation of the selected material model to place it properly in a variety of the developed ones. A discussion is restricted to the small displacement range, since it corresponds with the values observed in the mechanistic design approach.

Two main factors governing the neat bitumen response can be classified as rate- and temperature-dependency. The first one leads to the development of the material models accounting for its viscosity [6,7,26,27]. This behavior analysis is typically accompanied with the study on impact of the elastic [30] and plastic [31,32] part. These models usually have a clear mechanical interpretation as a combination of springs, dashpots and their connections (in parallel or in series). A dashpot type distinguishes between linear [26,33,34] and nonlinear [26,30,33] models. In the case of basic models built using such combinations, parameter determinations can be performed using physical interpretations of the selected model components. It is critical to the reliability of the model and more justified than alternative curve-fitting that is commonly used for the determination of some ad hoc model parameters.

In the present study, a linear viscoelastic Burgers model [26,33] is implemented. This is due to its feasibility for typical bitumen behavior modeling in the respective frequency range, efficient time-integration scheme and intuitive mechanical representation.

Temperature dependency of the binder phase is numerically modelled in a manifold manner [26,32,33]. One can use a variety of so-called shift factors to transfer the response obtained at the reference temperature to the real analysis temperature [26,33]. Alternatively, material parameters can be expressed in terms of the temperature [26]. Damage induced by the temperature changes was introduced to another model [32].

In the present study, a constant temperature is assumed for the sake of brevity. Thus, a single set of material parameters is necessary for a particular numerical test. No thermal effects, besides the model parameters valid only for a specific analysis temperature, are assumed.

The finite element analysis is performed using a standard displacement formulation (see the next chapter for details) with linear triangular elements. In our previous papers (see, e.g., [8]), higher-order approximation and multiscale analysis were also employed. For the sake of brevity, they are not used in this paper.

Summing up, a novel framework for the viscoelastic AC finite element analysis with a digitally reconstructed microstructure is presented in this study. The internal structure of the AC specimens is recognized by using high-quality digital images with their further processing. After the segmentation step and assessment of the aggregate boundaries, vector graphics are simplified in a controlled manner to facilitate finite element method computations. The Burgers material model is employed to describe the binder phase response, and aggregate is considered as linear elastic. The whole framework is tested on a set of numerical examples to confirm its numerical efficiency.

## 2. Materials and Methods

### 2.1. Specimens and Images

The specimens were prepared in the laboratory of the Chair of Highway, Railway and Traffic Engineering of Civil Engineering Faculty (Cracow University of Technology, Cracow, Poland). The asphalt mixture was AC 16, with a neat bitumen 35/50 and dolomite aggregate. From cylindrical specimens of a diameter base equal to 16 cm, a set of 5 cm thick slices were cut with a circular saw. The high-quality photos at the resolution of 24 Mpx were taken, from which idealized shapes were trimmed to be further processed.

### 2.2. Image Processing

**Figure 2:**
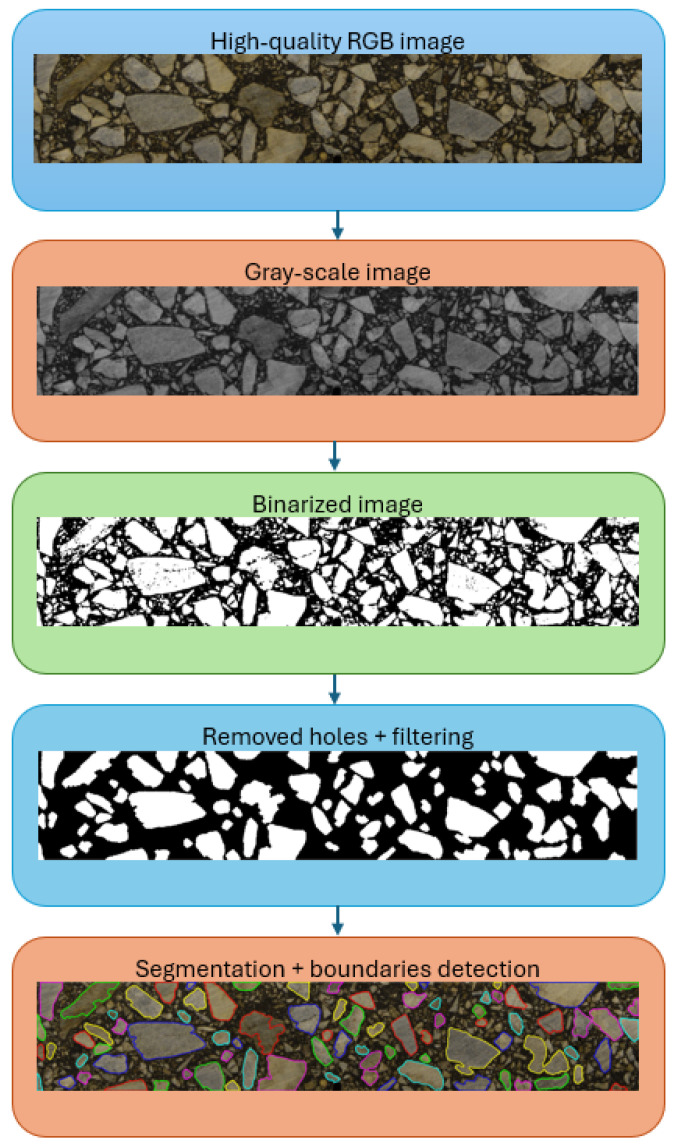
Digital image processing performed on AC specimen.

Specimen images are digitally processed in order to provide the input for the finite element analysis. In this study, we use the workflow proposed in [25]. Namely, a set of operations is performed to process a given image to a form ready for segmentation, i.e., independent image objects recognition. A scheme of this workflow is shown in Figure 2.

In general, the workflow presented in Figure 2 is common for most of the frameworks developed [14,21]. The differences consist of various specific numerical algorithms implemented in the consecutive steps of image processing. It should be clearly stated that there is no one unique combination of these algorithms, which exhibits its superiority for all kinds of asphalt mixture. In the case of AC, there are many aspects that influence effectiveness of the algorithms used: gradation curves, type of bitumen and aggregate, and variations in the crushed stone shapes, to mention only a few. Practically, it means that specific algorithms (or at least their parameters) should be adopted for the respective application.

Below, the main steps of the image processing are briefly discussed. Firstly, it is crucial to provide for the further processing specimens that are clean (e.g., no smeared bitumen is observed due to the sawing process) and free of dust. Specimens should also be well highlighted in order to not reduce the image quality due to possible local shadows.

In some studies, high-quality images are also subjected to the Gaussian filtering. This smears some small particles and exclude them from the further processing. An undesired effect of such an operation is, however, reducing the contrast between neighboring coarse aggregates. In the present study, no Gaussian filtering is used. Instead, a threshold value for the smallest particle of interest is defined at the further processing steps.

A red–green–blue (RGB) image is further transformed to the gray-scale image on the basis of the intensity parameter (see [25] for details).

Subsequently, the gray-scale image is binarized using the adaptive approach (locally in the neighborhood of the analyzed pixel), and the so-called “holes” are removed from the inclusions.

At this step, an erosion operation (diminishing the size of the inclusion by trimming its boundary) is performed, and the particles smaller than the user-defined threshold value are removed. A dilation operation is consequently performed to restore the previously reduced inclusion dimensions.

Segmentation of the aggregate particles is followed by their boundaries detection. In Figure 2, the exemplary final effect of the procedure described above can be observed.

Up to this step, image processing is performed using raster graphics. As it was mentioned in the Introduction, this is followed in this study by operations performed using vector graphics. In order to facilitate the finite element analysis, the AC microstructure geometry is simplified in a controlled manner, using the local geometry enhancement algorithm proposed in [25]. The first step is to scale the raster graphics to the physical dimensions. Pixel centers are considered as the line endpoints, and the microstructure geometry is described in terms of the vector graphics. A direct import of the geometry in such a form would lead to the generation of the overkill mesh of the extreme density. It was demonstrated in [25] that a reasonable geometry simplification can speed-up the computations by introducing only a very small additional modelling error.

The local geometry enhancement algorithm (see [25] for details) starts with the boundary being the output of the image processing. The first iteration of the algorithm is based on the quadrilateral spanned on the vertices being the 4 outermost points of the inclusion boundary. In the next iteration, the distances between the quadrilateral edges and the corresponding boundary points (edge endpoints) are measured. The points with the maximum distances constitute vertices of the new polygon, which is the iteratively corrected inclusion shape. In the limit, this algorithm reconstructs the geometry that is the output of the image processing. In this study, the iterative process is controlled by the inclusion area fit, i.e., iterations stop when the tolerance between the initial and updated inclusion geometry area is acceptable.

### 2.3. Problem Formulation

A strong formulation of the general viscoelasticity problem is recalled below. It is presented in a standard displacement form, in which the displacement field $u\left(x,t\right)$ is the sought unknown. Alternatively, mixed or discontinuous formulations can be employed [35].

Find a vector field of displacements u(x,t) such that

(1) $$\left\{\begin{array}{l} div\dot{\sigma }+\dot{X}=0\;\;\;\;\;\;\;\;\;\;\;\;\;\;\;\;\;\;\;\;\forall t,\;\forall x\in \omega_{i}\subset \Omega \\ \dot{\sigma }=C^{-1}\left[\dot{\varepsilon }\left(\dot{u}\right)-{\dot{\varepsilon }}^{*}\right]\;\;\;\;\;\;\;\;\forall t,\forall \;x\in \omega_{i}\subset \Omega \\ \dot{\varepsilon }=\frac{1}{2}\left[\nabla \dot{u}+{\nabla \dot{u}}^{T}\right]\;\;\;\;\;\;\;\;\;\;\;\;\;\;\forall t,\;\forall x\in \omega_{i}\subset \Omega \\ \varepsilon^{*}=\int_{0}^{t}J(t-\tau )\dot{\sigma }d\tau \;\;\;\;\;\;\;\;\;\;\forall t,\forall \;x\in \omega_{i}\subset \Omega \\ \begin{array}{c} \begin{array}{cc} +\mathrm{initial},\;\mathrm{boundary}\;\mathrm{and} & \end{array}\\ \begin{array}{cc} \mathrm{continuity}\;\mathrm{or}\;\mathrm{debonding}\;\mathrm{conditions} & \end{array} \end{array} \end{array}\right.$$

where a superimposed dot stands for a derivative with respect to time t, σ is the stress tensor, X is the field of (possible) body forces, C is the material parameters tensor, J is the function of creep compliance, $\varepsilon^{*}$ is the inelastic strain tensor, Ω is the whole analyzed domain, $\omega_{i}$ is the subdomains with continuous C, and τ is the integration variable. For the finite element analysis, the weak problem formulation is necessary. It is rewritten below.

Find the vector field of displacements $u\left(x,t\right)\in H_{0}^{1}\left(\Omega \right)+\hat{u}(x,t)$ such that

(2) $$\int_{\Omega }\varepsilon \left(v\right):C^{-1}\dot{\varepsilon }\left(\dot{u}\right)d\omega =\int_{\Omega }v\cdot \dot{X}\;d\omega +\int_{S_{\sigma }}v\cdot \dot{\hat{t}}ds+\int_{\Omega }\varepsilon \left(v\right):C^{-1}{\dot{\varepsilon }}^{*}d\omega \;\forall t,\;\forall v\in H_{0}^{1}\left(\Omega \right)$$

where $S_{\sigma }$ denotes the boundary part with the Neumann boundary conditions imposed, v denotes a test function, $\hat{t}$ is the imposed traction, $\hat{u}$ is the Dirichlet boundary condition (known displacement field), and $H_{0}^{1}$ is the Sobolev space of functions satisfying homogeneous Dirichlet boundary conditions.

Linearization of Equation (2) leads to the incremental formulation.

Find the vector field of displacement increments $\Delta u\left(x,t\right)\in H_{0}^{1}\left(\Omega \right)+\Delta \hat{u}(x,t)$ such that

(3) $$\int_{\Omega }\varepsilon \left(v\right):C^{-1}\Delta \varepsilon \left(\Delta u\right)d\omega =\int_{\Omega }v\cdot \Delta X\;d\omega +\int_{S_{\sigma }}v\cdot \Delta \hat{t}ds+\int_{\Omega }\varepsilon \left(v\right):C^{-1}\Delta \varepsilon^{*}d\omega \;\;\;\forall v\in H_{0}^{1}\left(\Omega \right)$$

**Figure 3:**
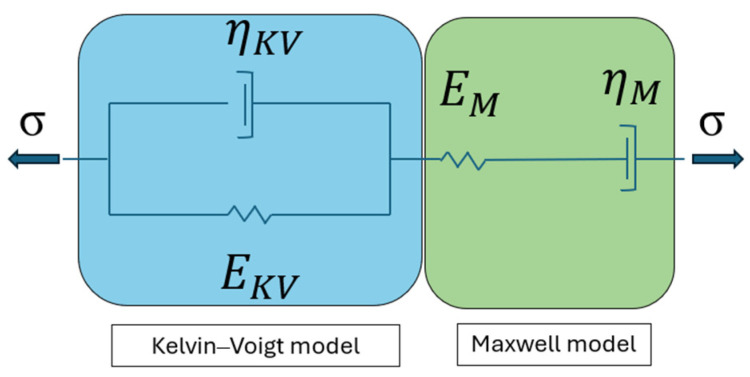
Scheme of the Burgers material model—basic form.

In this study, the Burgers material model is employed for $\Delta \varepsilon^{*}$ definition. Its mechanical interpretation is shown in Figure 3.

It comprises the Kelvin—Voigt and Maxwell models joined in series. The Kelvin—Voigt element is used to model a delayed viscoelastic material response, a spring in the Maxwell element is used to model an instantaneous elastic response, whereas the damper is used to model the irrecoverable deformation. In the literature [26,33], the generalized version of the Burgers model is described as a combination comprising, besides the Maxwell element, a number of the Kelvin–Voigt elements joined in series.

Recalling from [33], an additive decomposition of $\Delta \varepsilon^{*}$ into the elastic $\Delta \varepsilon_{EL}$, viscoelastic $\Delta \varepsilon_{VE}$ and viscous $\Delta \varepsilon_{V}$ terms can be expressed using Equations (4)–(6).

(4) $$\Delta \varepsilon_{EL}=C\Delta \sigma$$

(5) $$\Delta \varepsilon_{VE}=\sum_{i=1}^{N}\left\{\varepsilon_{VE,t-\Delta t}^{i}\left(e^{\frac{-\Delta t}{\tau_{i}}}-1\right)+\Delta te^{\frac{-\Delta t}{2\tau_{i}}}C_{VE}^{i}\left(\sigma_{t-\Delta t}+\frac{\Delta \sigma }{2}\right)\right\}$$

(6) $$\Delta \varepsilon_{V}=\Delta tC_{V}\left(\sigma_{t-\Delta t}+\frac{\Delta \sigma }{2}\right)$$

In Equations (4)–(6), t−Δt refers to the previous time instance, N is the number of the Kelvin—Voigt elements, $\tau_{i}$ is the retardation time of the *i*th Kelvin—Voigt element, and $C_{V}$ and $C_{VE}$ are constitutive matrices of the viscous and viscoelastic element, respectively [33].

### 2.4. Numerical Implementation

**Figure 4:**
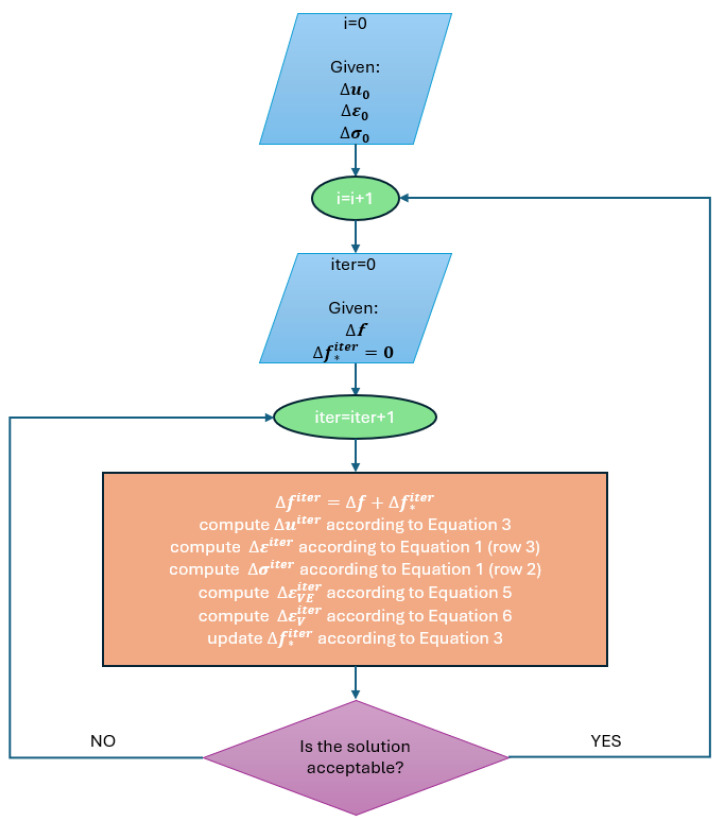
Time-stepping algorithm of the Burgers viscoelastic model.

A transient finite element analysis is necessary in the case of the linear viscoelastic Burgers model. A time-stepping algorithm in a form implemented in this study is schematically presented in Figure 4.

The analysis requires initial conditions. Typically, $\Delta u_{0}$, $\Delta \varepsilon_{0}$ and $\Delta \sigma_{0}$ are equal to zero. In Figure 4, the index “*i*” refers to the i-th time instance. Their total number is specified by the user accounting, e.g., for the load program.

Solution obtained for the first iteration (*iter* = 1) is in fact a linear elastic solution due to the fact that $\Delta f_{*}^{iter}$ (which stands for the contribution to the load vector because of the inelastic strain occurrence; see Equation (3)) is initially equal to zero. Inelastic strain increments $\Delta \varepsilon_{VE}$ and $\Delta \varepsilon_{V}$ are computed in terms of the stress increment Δσ. They are subsequently used to compute updated contribution to the load vector $\Delta f_{*}$, which is used to solve the problem described by Equation (3) and obtain the updated solution increment Δu. This process is iteratively repeated until the solution is acceptable. In the present study, two measures are controlled. Namely, the relative error between the subsequent displacement and total strain increment iterations. In the examples solved for the illustrative purposes and presented in the next section, a threshold value of 1% is used for both of these measures. The iterations are repeated for every time instance during the whole analysis period T.

Some numerical aspects are additionally to be clarified:

- The solution to Equation (3) requires only the right-hand-side vector update (due to $\Delta f_{*}$ modification), whereas the stiffness matrix (left-hand-side) remains constant for all iterations and time instances, which is computationally effective;
- Linear shape functions are used for the triangular finite elements in the examples solved in the next section;
- Application of the linear shape functions to approximate the solution within each triangular element implies that strains are constant within whole element (due to constant shape function derivatives)—consequently, numerical integration over the finite elements is also very fast (see Equation (3));
- Sparse matrix operations are used to speed-up the computations—together with the constant stiffness matrix (left-hand-side matrix of Equation (3)), it significantly facilitates the finite element analysis.

## 3. Results

In this section, two illustrative examples are solved in order to present the proposed approach. The first of them is a typical creep test performed on bitumen specimens. Herein, the idealized asphalt concrete sample is analyzed accounting for the material heterogeneity. The second example reconstructs the compression test performed on the asphalt concrete sample. For both tests, the aggregate is numerically modelled as the linear elastic and a perfect bonding is assumed between aggregate particles and surrounding bitumen, which is modelled using the Burgers viscoelastic model. In numerical implementation, the evolution of the total strain is equal to the evolution of the linear elastic strain in aggregate and additively computed strain (Equations (4)–(6)) in bitumen. Practically, the elementwise contribution to the global vector $\Delta f_{*}$ is computed only in the finite elements belonging to the bitumen phase and subsequently assembled.

The assumption on the plane strain is made for both examples. Also, the same image is processed in order to reconstruct the AC microstructure.

### 3.1. Creep Test

**Figure 5:**
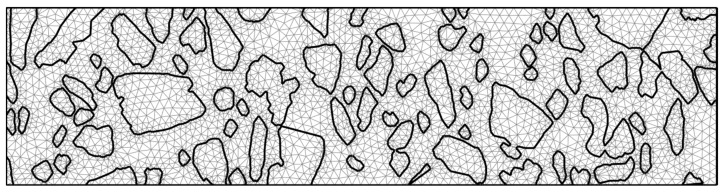
The simplified AC microstructure and the generated finite element mesh.

The input image of the AC specimen and its processed versions are presented in Figure 2. In Figure 5, the output image with the simplified AC microstructure geometry and the finite element mesh is shown. Specimen dimensions are 3 cm × 12 cm.

Boundary conditions for the creep test are used to reconstruct a tensile test with the load of the constant intensity subjected for a selected time period. In this numerical test, the symmetry boundary conditions are imposed at the right-hand-side (horizontal displacement component is equal to 0) and the bottom edges (vertical displacement component is equal to 0, therein), while a tensile load is applied to the right-hand-side edge. Analysis period is equal to 60 s, the load of intensity 1.5 kN/m is applied for the first 15 s. After that, it is removed. The time step used for the integration in time is equal to 0.1 s. Material data is specified in Table 1 for the aggregate and in Table 2 for the bitumen using the available literature data (c.f. [36]).

**Figure 6:**
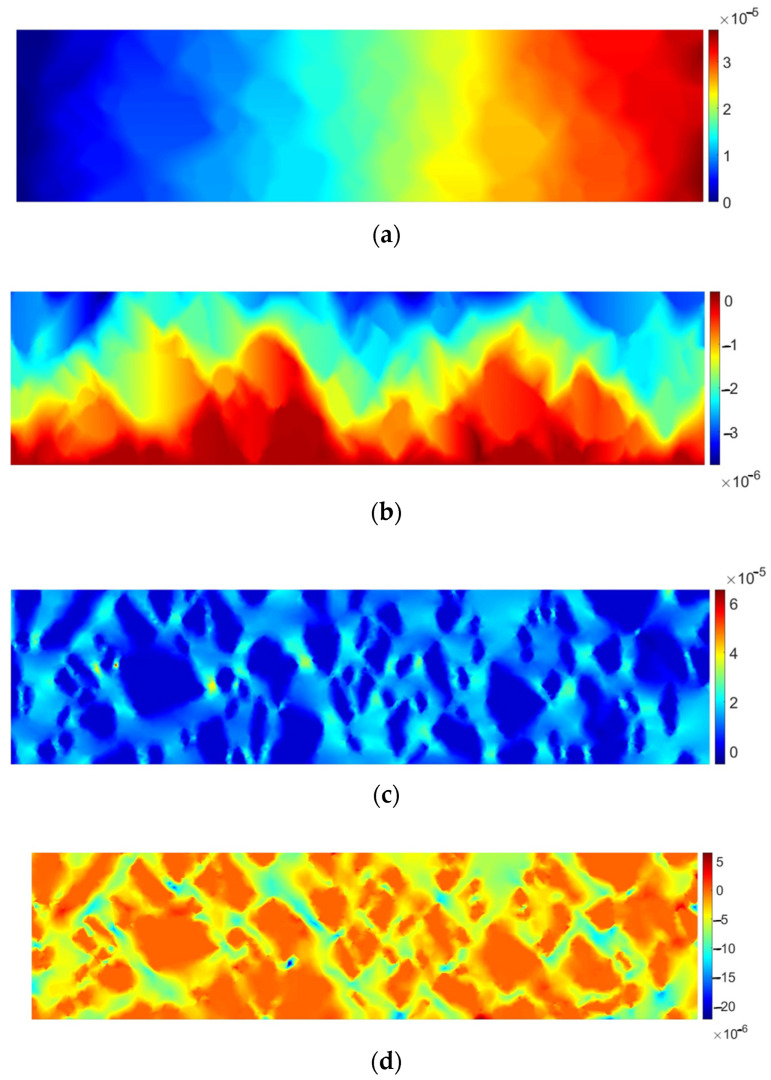
AC specimen elastic response: (**a**) horizontal displacement *u_x_* [m], (**b**) vertical displacement *u_y_* [m], (**c**) tensor strain component ε_xx_ [-], (**d**) tensor strain component ε_yy_ [-].

In Figure 6, the specimen response in the elastic range is presented.

**Figure 7:**
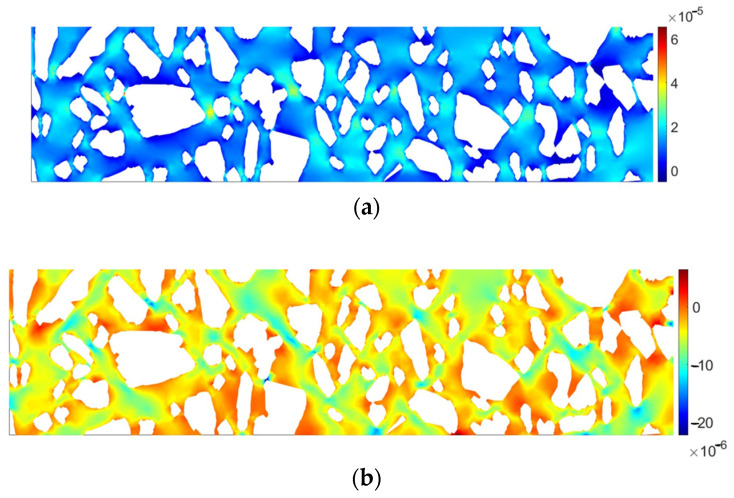
Strain distribution within AC matrix: (**a**) tensor strain component ε_xx_ [-], (**b**) tensor strain component ε_yy_ [-].

It can be clearly observed that the material response is not very similar to the homogeneous domain. A dominant displacement component *u_x_* would be in that case linear, which is not observed in Figure 6c. Linear behavior is also disturbed for *u_y_* due to a strong heterogeneity of the domain (Figure 6b). In Figure 6c,d it can be observed that strains in the inclusions are almost equal to zero, whereas greater values are present in the matrix (bitumen). In order to illustrate this observation, the quantities ε_xx_ and ε_yy_ are plotted separately for the matrix in Figure 7.

**Figure 8:**
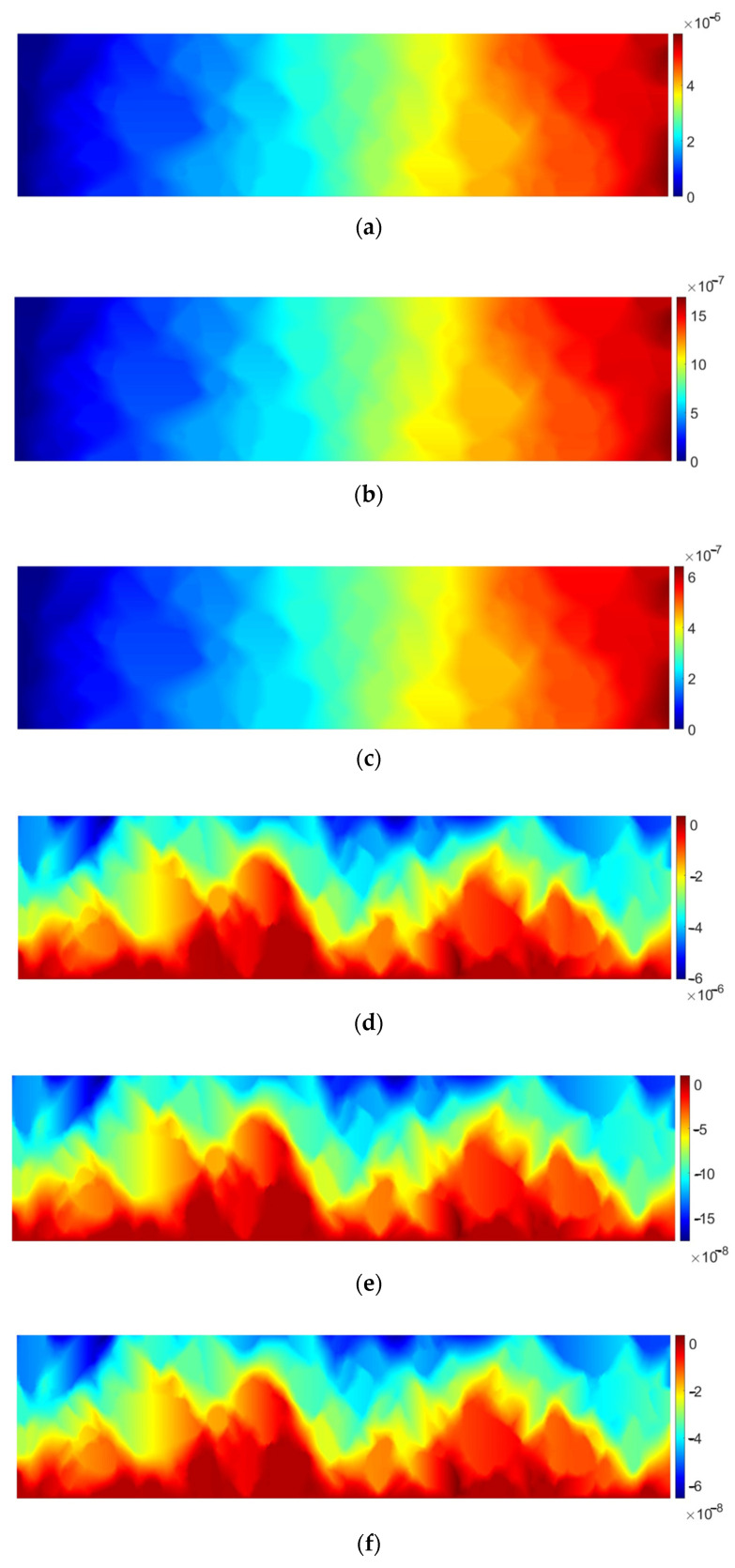
AC specimen viscoelastic response: (**a**) horizontal displacement *u_x_* [m] at *t* = 15 s, (**b**) horizontal displacement *u_x_* [m] at *t* = 30 s, (**c**) horizontal displacement *u_x_* [m] at *t* = 60 s, (**d**) vertical displacement *u_y_* [m] at *t* = 15 s, (**e**) vertical displacement *u_y_* [m] at *t* = 30 s, (**f**) vertical displacement *u_y_* [m] at *t* = 60 s.

The above observations are also valid for the viscoelastic analysis. In Figure 8, for the sake of brevity, displacements for three selected time instances are shown: at *t* = 15 s (just before the load removal), at *t* = 30 s, and at the end of the analysis period (*t* = 60 s).

In Figure 8, the evolution of displacement components is shown. At *t* = 15 s, the elongation exhibits its maximum value. It should be noted that the obtained values are approximately 45% greater than those obtained in the elastic analysis (see Figure 6). At *t* = 30 s and *t* = 60 s, the values drop down due to the load removal. What is prominent for the viscoelasticity is that there is a delayed material response observed (modelled by the spring and dashpot joined in parallel). Displacement component values drop down asymptotically, but even at *t* = 60 s, they are not equal to zero—this phenomenon is modelled by the dashpot in the Maxwell element of the Burgers model.

**Figure 9:**
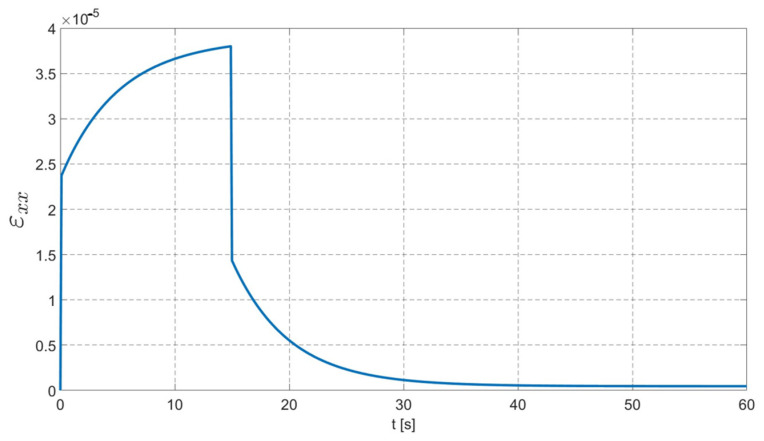
Evolution of ε_xx_ component in time.

In Figure 9, the evolution of the dominant tensor strain component ε_xx_ is plotted for the finite element located in the middle of the right-hand-side edge.

In Figure 9, one can observe a typical creep curve of the Burgers material. There is an instantaneous elastic strain clearly visible. Subsequently, there is a creep phenomenon observed, i.e., the evolution of the strain with a constant stress. After *t* = 15 s, when the maximum value is exhibited, there is also an instantaneous response observed as a dramatic drop in ε_xx_ value (modelled by the spring in the Maxwell part of the Burgers model)_._ Finally, an asymptotic drop can be seen. It should be noted that there is an irrecoverable part of the total strain, which accumulates in the case of the real-life conditions and repeatable loads. It is evidently seen as the evolution of the rutting process of the asphalt pavement structure.

### 3.2. Compression Test

In this test, the same microstructure geometry and material data are used. Boundary conditions are as follows: the bottom edge is fixed (both displacement components are equal to zero), and the compressive load is applied to the top edge. Imposed boundary conditions are to reflect a typical performance conditions of the asphalt pavement layer. Thus, the vertical displacement component and its evolution in time are of the particular interest.

**Figure 10:**
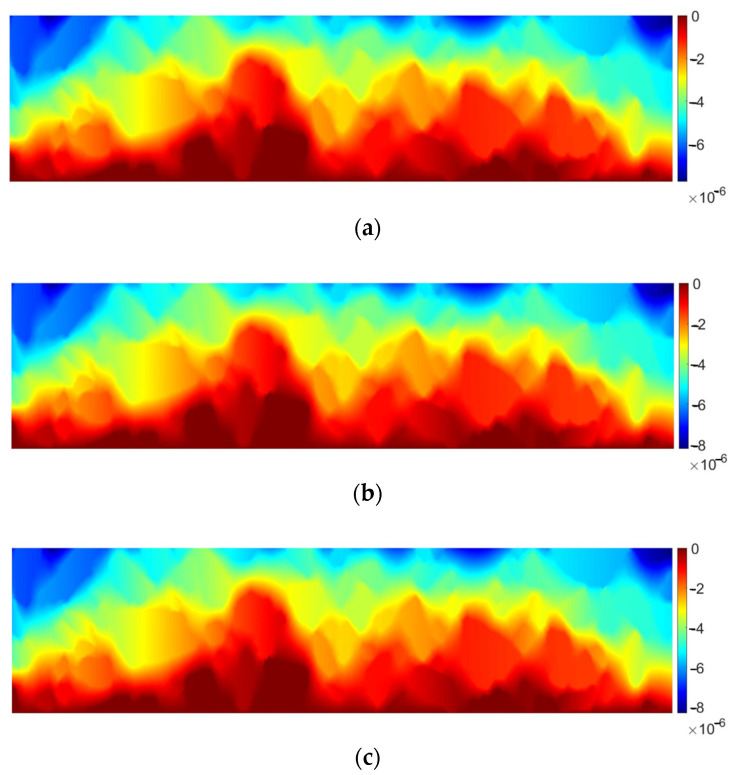
Evolution of *u_y_* [m] in time plotted for time instances: (**a**) *t* = 10 s, (**b**) *t* = 20 s, (**c**) *t* = 30 s.

The load intensity is equal to 0.8 kN/m in this test. It is constant for the whole analysis period that is equal to 30 s. The time step used for the integration in time is set as 0.1 s. In Figure 10, the evolution of the vertical displacement component in time is shown with the interval of 10 s.

As it can be observed in Figure 10a–c, the load program (or load duration in real-life asphalt pavement performance) influences the final material response. The results presented in Figure 10a–c coincide with those shown in Figure 9. It can be observed that, for the constant load, Burgers material exhibits an asymptotic behavior, i.e., a nonlinear displacement/strain increase is obtained. The rate of this process decreases with time. In Figure 9, the strain asymptotic limit is approximately equal to 3.8 × 10^−5^. In Figure 10a–c, it can also be observed that the displacement rate decreases with time, and the vertical displacement approaches −8.45 × 10^−5^ m. The study on the load duration and its impact on the bituminous material performance has been studied in the literature [26,27,36].

## 4. Discussion

The results presented in Section 3 are to illustrate a general framework for the finite element AC analysis that accounts for the internal structure of the material. In this study, a 2D case was studied and the high-quality digital image processing was used for the microstructure geometry recognition. It can be regarded as the initial study on the XRCT scans processing and the prospectives of the geometry recognition, followed by its further controlled simplification. Benefits demonstrated for a 2D case (c.f. [25]) can be obtained for a full tridimensional finite element analysis due to a substantial NDOF reduction.

In this paper, the applicability of the image processing scheme is presented in the viscoelastic analysis of the AC specimen. Reliable methods for computation speed-ups are of particular interest in the case of transient problems (as described by Equation (3)), where large systems of algebraic equations are solved at each time step.

Further research effort is to propose an effective algorithm for the XRCT scans processing that leads to a simplified (in a controlled manner) AC microstructure geometry. Other material models are also to be tested. A cohesive zone model is to be applied for the aggregate-bitumen interaction modeling. Additionally, the multiscale finite element analysis will be applied in order to obtain the computation speed-up with a negligible solution error.

## 5. Conclusions

Summing up, the following conclusions can be drawn:

- High-quality digital image processing is an effective method for 2D asphalt concrete microstructure recognition. The output of this technique application can be utilized in a manifold manner. Successful studies linking 2D images with a 3D microstructure characterization can be found in the literature [21]. Alternatively, the numerical apparatus developed for the high-quality digital image processing can be used for the XRCT scans processing, which allows for a 3D AC microstructure recognition;
- Controlled simplification of the microstructure geometry facilitates the finite element analysis. This approach can be particularly profitable in the context of the 3D AC microstructure reconstruction on the basis of the set of XRCT scans;
- Viscoelastic finite element analysis using the Burgers model presents the overall AC specimen behavior with good accuracy. Adopting the cohesive zone model for the aggregate–binder interface would also increase the reliability of the analysis. A number of other material models for the bitumen should be also studied, e.g., in the case of the large displacements expected;
- Further FEA speed-up can be obtained using the multiscale/homogenization method. Such analysis should be accompanied with an error analysis to verify the reliability of the proposed approach.

## Figures and Tables

**Table 1:** Material data for aggregate.

Property	Value	Unit
Young modulus E	75,000	[MPa]
Poisson ratio ν	0.3	[-]

**Table 2:** Material data for bitumen.

Property	Value	Unit
Young modulus E_M_	70	[MPa]
Young modulus E_KV_	120	[MPa]
Poisson ratio ν	0.3	[-]
Poisson ratio equivalent ν_M_	0.3	[-]
Poisson ratio equivalent ν_KV_	0.3	[-]
Viscosity η_M_	60,000	[MPa s]
Viscosity η_KV_	600	[MPa s]

## Data Availability

Data are contained within the article.
